# The Ability to Run in Young People with Cerebral Palsy before and after Single Event Multi-Level Surgery

**DOI:** 10.3390/jpm11070660

**Published:** 2021-07-14

**Authors:** Roman Rethwilm, Harald Böhm, Leonhard Döderlein, Peter A. Federolf, Chakravarthy U. Dussa

**Affiliations:** 1 Department of Sport Science, University of Innsbruck, 6020 Innsbruck, Austria; peter.federolf@uibk.ac.at; 2 Orthopedic Children’s Hospital Aschau GmbH, 83229 Aschau im Chiemgau, Germany; h.boehm@bz-aschau.de (H.B.); c.dussa@bz-aschau.de (C.U.D.); 3 Orthopedic Clinic Aukamm, 65191 Wiesbaden, Germany; leonharddoederlein@gmx.de

**Keywords:** cerebral palsy, locomotion, running, SEMLS, 3D gait analysis

## Abstract

The objective of the study is to identify and evaluate possible factors that influence the ability to run before and after single event multi-level surgery (SEMLS). Young patients (6–25 years) with spastic cerebral palsy (GMFCSI-II) were retrospectively included. Type and number of surgical procedures, time for recovery and 3D gait analysis variables were analyzed with respect to the ability to run. In total, 98 patients (38 females; 60 males) who received SEMLS (12 years, SD 3.4) were included and compared to a control group of 71 conservatively treated patients. Of 60 runners pre-surgery, 17 (28%) lost the ability, while gained in 8 of 38 (21%) non-runners. The number of surgical procedures was a significant predictor and those who lost their ability to run had significantly more (mean = 5.9, SD = 1.7), compared to the patients who gained the ability (mean = 3.5, SD = 0.9). Further, pre-surgical function (e.g., gait speed) was significantly different (*p* < 0.001). Pre-surgical function and the number of surgical procedures seem to play an important role for the gain or loss of the ability to run after surgery. Caution is warranted in patients with lower pre-surgical function and the ability to run, as they seem at a higher risk to lose the ability.

## 1. Introduction

The ability to run is an important and fundamental movement skill. It is especially crucial for children and young people for playing and engaging in sports. Studies have shown that higher functionality, including running, positively influences the integration and participation of children with cerebral palsy (CP) [1,2,3]. Furthermore, being physically active and participating in adapted sports has been shown to increase the quality of life [4] and is known to play a crucial role in preventing or reducing secondary health impairments based on inactivity [5].

The major goals of orthopedic surgery in CP are to improve or facilitate function by restoring muscle lever arms and reducing contractures and spasticity [6]. Surgical interventions were shown to improve walking function, assessed by instrumented gait analysis [7]. However, evidence suggests that walking performance might not necessarily predict running capability [8] and some authors propose that the pathological posture of CP, especially the equinus foot, is more congruent with running posture than walking [9]. This suggests that walking gait parameters alone might fail to deliver a complete picture of motor function; furthermore, it is unclear if procedures which are beneficial for walking ability are equally beneficial for running. Therefore, the ability to run might be interesting as a further outcome measure when evaluating and quantifying the outcome of surgical interventions.

The ability to run is already part of several functional clinical assessment tools such as the Gross Motor Function Classification System (GMFCS) [10] and the Gross Motor Function Measure (GMFM) [11,12]; however, it becomes diluted within the overall score. As a result, patients who are able to run can reach the same GMFM score or GMFCS level as those who are unable to run. For example, GMFCS level II includes runners and non-runners. Running, however, is an important motor functional skill and may be underestimated within the mentioned measures of function.

Multiple factors may affect the ability to run after orthopedic surgery. The number of surgical procedures and the type of surgery are likely to influence the ability to run after surgery. For example, specific bony procedures, such as femoral or tibial derotation osteotomies, might be beneficial for the ability to run since they improve the muscular lever arms [13] and, therefore, enhance the muscle force output that is needed for running. Furthermore, in a previous study we identified rectus femoris muscle spasticity to be a negative predictor for the ability to run, whereas, gastrocnemius spasticity was a positive predictor [14]. Consequently, muscle tenotomies of the rectus femoris might be beneficial and calf muscle lengthening could be detrimental. Despite the effects of different surgical procedures, the pre-surgical function is an important aspect. The pre-surgical gait speed, as a measure of function, has already been identified as a predictor for the outcome of surgery [15]. Furthermore, the post-surgical measurement time point might also be an important factor, since the time for recovery is crucial to regain strength and function [16]. With respect to recovery time, the gait speed was often found to be still reduced 1 year after multi-level surgery but increased with advancing time [7], which might also translate to running ability.

Using objective measures from clinical gait analysis in conjunction with the ability to run as functional assessment tools could help to further personalize the surgical treatment of people with CP. Hence, gaining insight into factors that contribute to the presence of the ability to run before and after orthopedic surgery is another step towards personalized medicine and might help clinicians to improve their decision making and to better inform about patient expectations.

Therefore, the objective of the current study was to quantify the presence of the ability to run in young patients with CP before and after orthopedic surgery and to identify possible factors contributing to the gain or loss after surgery. The factors include the pre-surgical function quantified by the gait speed, Gait Profile Score (GPS) and GMFCS. Furthermore, surgical procedures and their number in one SEMLS and changes in walking speed, GPS and BMI, as well as the time to follow-up, were investigated.

## 2. Materials and Methods

The study was retrospectively conducted including young patients with spastic CP, who underwent 3D gait analysis before and after orthopedic multi-level surgery and a control group of patients who had no previous surgery and were only conservatively treated between two measurements. All patients and/or guardians provided written consent and the local ethics committee (Bavarian State Medical Association) stated that their approval was not required.

Patients included were at least 6 years old at pre-measurement and not older than 25 years at follow-up. Both, uni- and bilaterally involved patients with CP (GMFCS I-II) were included. Accordingly, all patients were able to walk freely without any assistive devices preoperatively [10]. The recovery time to follow-up had been at least 12 months. Exclusion criteria were additional syndromes, lack of compliance or comprehension of the running task. Obese patients according to the age dependent body mass index (BMI) thresholds recommended by the WHO [17] were also excluded.

All patients were referred to instrumented 3D gait analysis as part of the clinical procedure by a physician for diagnostic purposes, decision making or evaluation. The gait analysis was conducted barefoot at a self-selected walking speed, using an eight-camera Vicon MX system (Vicon Inc., Oxford, UK) on a 13m walkway. Additionally, patients were asked to run at a self-selected jogging pace or, if not possible, at their fastest pace of locomotion. The ability to run was defined by the presence of a double float phase [18].

Single event multi-level surgery (SEMLS) was defined as surgery involving two or more anatomical levels at the lower extremity with two or more soft-tissue or bony surgical procedures during a single session [19].

The type and number of orthopedic procedures was counted and evaluated for every patient. The individual surgery type was counted only once, even when it was conducted bilaterally. However, the number of surgical procedures accounts for the sum of all procedures, except for foot surgeries, which were counted collectively as bony or soft-tissue foot surgeries. Rarely performed procedures were grouped as “other bony” or “other soft-tissue” surgeries.

Additionally, the following parameters were compiled: follow-up time, defined as the time span between the date of surgery and post-surgical gait analysis; the age and walking Gait Profile Score (GPS) [20] before surgery; and differences between pre- and post-measurements in non-dimensional walking speed [21], GPS and percent of BMI deviation.

Patients were divided into four groups: non-runners pre- and post-surgery (run00), non-runners pre-surgery who gained the ability to run (run01), runners pre-surgery who lost their ability (run10) and runners who retained their ability to run (run11). As reference, a control group of patients who could all run and were treated conservatively was included.

### Statistical Analysis

Kruskal–Wallis tests with Mann–Whitney U post-hoc tests were used for group comparisons due to small and unequal sample sizes and failed assumptions for one-way ANOVA. To account for multiple testing, the Benjamini–Hochberg procedure was used to control for a false discovery rate (FDR). *p*-values were deemed significant with *p* < 0.05 and an FDR < 0.1. The control group was balanced using the optimal full propensity score matching and weighting [22], based on the pre-age, pre-GPS and follow-up time. The weighting factor was used in all statistical tests involving the control group. Furthermore, two binary logistic regressions were calculated: one to determine the influence of the number of procedures on the likelihood of gaining the ability to run in patients who were unable to run pre-surgery (run00/run01), and the second to determine how the number of procedures affects the likelihood of losing the running ability within patients who were able to run pre-surgery (run10/run11).

RStudio (RStudio, Inc., Boston, MA, USA) was used for the propensity score matching and follow-up tests using the packages “MatchIt” and “Survey” and SPSS Statistics (IBM Corp., New York, NY, USA) for the binary logistic regressions.

## 3. Results

The inclusion criteria were met by 98 young patients (38 females; 60 males) with surgery and 71 conservatively treated control patients (28 female; 43 male). The pre-surgery mean age was 12.0 years (3.4 SD), ranging from 6 to 21 years, and post-surgery mean age 14.3 years (3.5 SD), ranging from 7 to 24 years. The control group mean pre-age was 9.9 years (2.6 SD) (range: 6–17 years) and 12.3 years (2.7 SD) (range: 8–19 years) at the follow-up. Anthropometrics are displayed in Table 1. After adjusting the control group age using weights, the control group mean pre-age was 11.7 years (3.0 SD) and no significant differences in the mean pre-age nor the mean time to follow-up were found between the groups (Table 2).

The ability to run was present in 61% (*n* = 60) pre-surgery and 52% (*n* = 51) post-surgery, where 21% (*n* = 8/38) gained the ability and 28% (*n* = 17/60) lost the ability after surgery. In the control group, only 1 of the 71 conservatively treated patients lost the ability to run due to an equinovarus.

The distribution of GMFCS level, involvement (uni- or bilateral CP) and if the patients received surgery on both legs, descriptively differed between the groups (Table 1). These differences, however, existed mainly between patients who could not run and patients who did not lose the ability (run00/run11), while those who gained or lost the ability (run01/run00) showed a similar distribution. The run10 patients were mostly (82%) classified as GMFCS II, whereas, in the run11 group, only 58% were GMFCS II. Furthermore, the run10 group had more often bilateral CP and accordingly, had more often surgery on both legs.

A more comprehensive description of precondition and functional status of the patient groups is the pre-surgical walking speed and GPS (Table 2). The groups who could run post-surgery (run01/run11) had less gait deviations compared to those who could not run post-surgery. However, statistically significant differences were only observed in the pre-walking speed between run00/run10 and run11, as well as run00 and control. The functional status, expressed by the non-dimensional gait speed, showed that those who lost the ability and those who did not gain the ability already walked significantly slower pre-surgery, compared to those who retained running. Those who gained the ability were also slower despite of having almost the same non-dimensional gait speed as the run10 group (run01 = 0.3734 ± 0.0512 vs. run10 = 0.3727 ± 0.0486).

**Figure 1:**
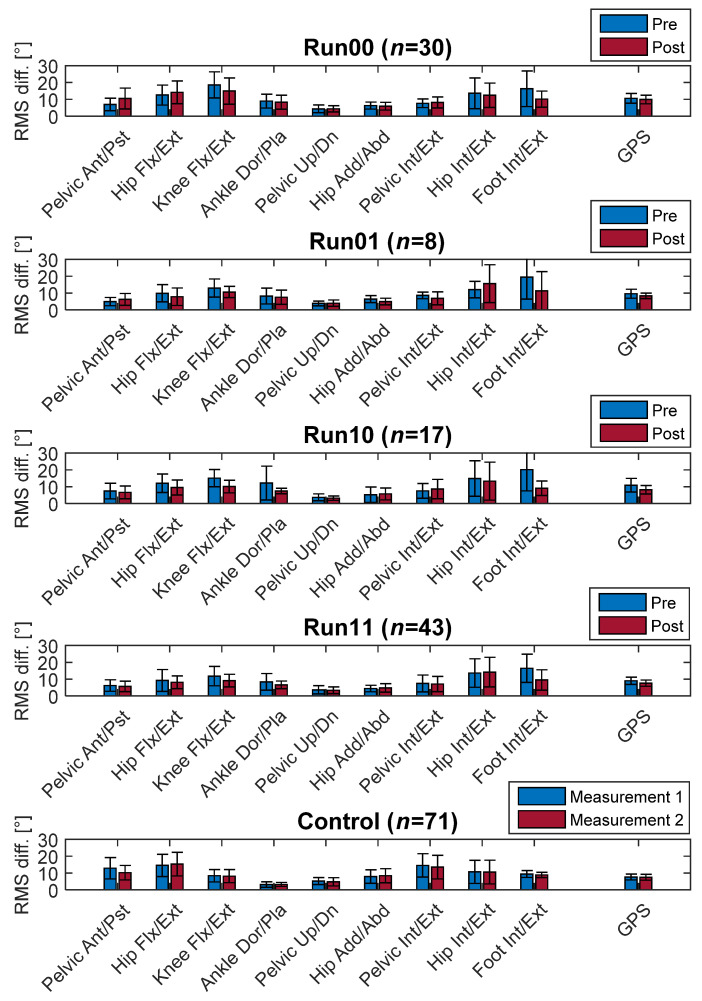
Movement analysis profile (MAP) and GPS changes (mean and SD). Non-runners (run00); ability gained (run01); ability lost (run10); runners (run11); RMS difference from norm (degree).

Post-surgical GPS improvements were observed in all groups. However, these changes were not significant. Interestingly, the run10 group counted the most improvements, despite losing the ability to run. A more detailed view of the joint angle deviations is depicted in Figure 1, showing the movement analysis profile (MAP).

The gait speed difference from pre- to post-measurement was significantly different between groups. The run01 group was the only group with a gait speed increase, being significantly different from all the other surgically treated patient groups, all showing a decline. This decline was also significantly different to the control group whose speed remained almost the same.

The number of surgical procedures was significantly different between the groups. These differences are clearly between the groups who could not run post-surgery (run00/run10) and those who could (run01/run11). The latter groups had significantly less surgical procedures as part of the SEMLS (Table 2). Additionally, in the logistic regression, the number of surgical procedures was a significant predictor for losing running (*p* < 0.001) or not gaining the ability post-surgery (*p* = 0.034). With every procedure, the probability of gaining the ability was reduced by a factor of 0.38 (95% CI: 0.16–0.93) in the pre-surgery non-running groups (run00/run01), and in the running groups (run10/run11) every additional procedure increased the chance of losing the running ability by a factor of 2.9 (95% CI: 1.64–5.20).

**Figure 2:**
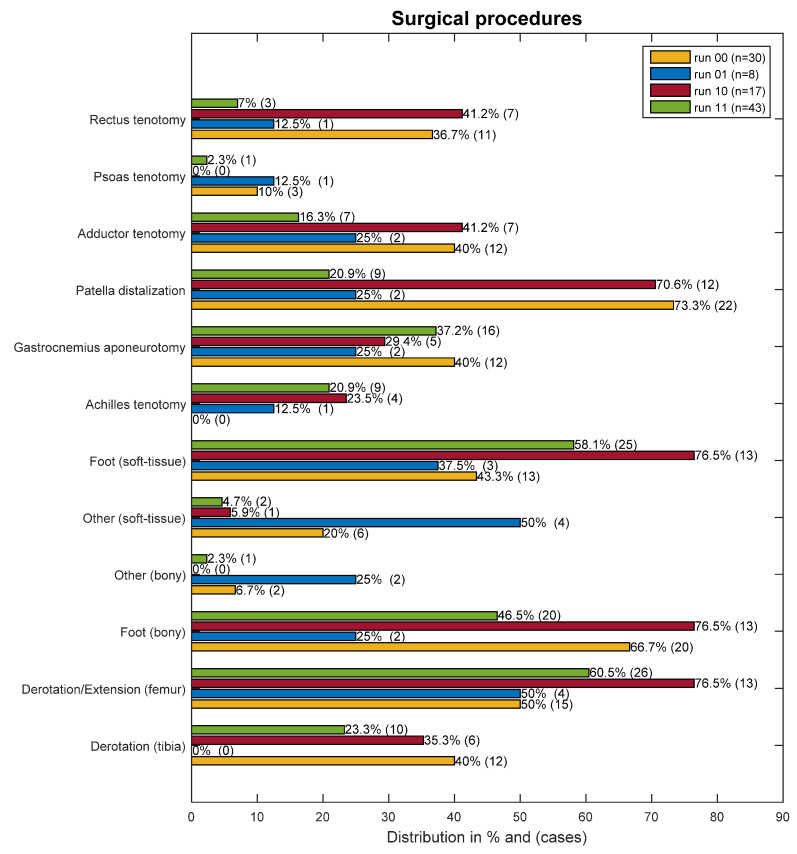
Overview of the surgical procedures in percent and number of incidences.

The relative prevalence of surgical procedures in the different groups is shown in Figure 2.

The post-surgical BMI increased only in the run11 and run00 groups, however, standard deviations were high, and no meaningful statistical differences were found.

## 4. Discussion

Running is a more demanding movement skill than walking and only mildly affected people with CP (GMFCS I-II) are able to run. This study is, to our knowledge, the first to investigate how SEMLS might influence the ability to run. We found that the ability was newly gained in 21% of the patients who could not run pre-surgery and lost in 28% of the patients who could run previously, while very seldomly lost (1.4%) in a conservatively treated reference group. Furthermore, the study revealed that the precondition and pre-surgical function likely influenced the chance of being able to run. Additionally, and interrelated was the number of surgical procedures identified as a significant predictor for the ability to run post-surgery.

The precondition appears to be one aspect influencing the ability to run. Even though, the involvement (hemi- or bilateral CP) was comparable between the patients who gained or lost the ability to run, having one well-functioning leg has been found to positively influence the presence of running ability [14]. In general, the involvement distributions, as well as the GMFCS level distributions, indicate that there is a critical transition area comprising pre-surgical “good” non-runners and “poor” runners, who are especially susceptible to change. For example, in group run10, most of the patients were classified as GMFCS II (82%), had bilateral CP (82%) and surgery to both legs (59%), while group run11 comprised fewer GMFCS II (58%), were less often bilaterally affected (63%) and had fewer bilateral surgeries (30%), showing obvious differences in the severity of the disease.

Another important factor is the pre-surgical function expressed by the GPS and gait speed. The patients who lost the ability to run had more severe gait deviations pre-surgery compared to the patients who gained the running ability. Therefore, pre-surgical gait function and identifying patients at the verge of losing the ability to run should be considered when planning a SEMLS. Despite the gait speed between these groups being almost identical, evident differences can be observed in the groups who did not change, drawing a clear line between the patients who are unlikely to gain or lose the ability. These results are in line with another study, who found that walking speed was a predictor for a positive surgery outcome [15].

GPS changes from pre- to post-measurement were not significantly different. Nevertheless, all groups had gait deviation improvements. Interestingly, the patients who lost the ability to run had the highest GPS improvements, which could indicate that despite the improvements in the joints angle deviations (i.e., MAP), the neuro-muscular system needs to adapt to the new situation caused by the surgery.

The minimum recovery time to follow-up was at least 12 months, with a mean above 20 months. However, longer recovery times may be warranted especially for patients who are at the verge of being able to run pre-surgery. One study, for example, found significant functional improvements between 1 and 2 years after SEMLS [23] and another found that the walking speed is often reduced or unchanged 1 year after surgery but increased at later follow-up time points [7]. The impression that in some cases longer recovery times are needed is supported by a few single cases in our clinic, where the ability to run was regained in the second or third follow-up measurement. However, more data and long-term results are needed to determine if the ability is lost temporarily or permanently.

In the current study, the walking speed was reduced after the surgery in all groups, except in the group who gained the ability and in the control group. When evaluating gait speed, changes in the results need to be judged in respect to the fact that there is a natural decline in walking function in CP [24]. Therefore, small gait speed improvements or no changes might still be a favorable outcome. However, the unchanged gait speed in the control group and only a single case in this group, where the ability to run was lost, indicates that gait deterioration might not be as prevalent in younger people with CP as in adults [24].

The number of surgical procedures received within the SEMLS is likely intertwined with the pre-surgical function. Not only is this depicted by the clear group differences between the post-surgical runners and non-runners, but also by being a significant predictor for the ability to run post-surgery. Despite a likely correlation with the number of deformities and gait pathologies that need surgical treatment, it also underlines the importance of careful planning and of a risk–benefit assessment.

The analysis of the influence of specific procedures is very difficult due to the diversity of surgical procedures within SEMLS. Nevertheless, in planning a surgery, it should be considered that some procedures might be more and others less beneficial for the ability to run. Gastrocnemius spasticity, for example, has been identified as a positive predictor for running ability [14], and from a biomechanical perspective, a lengthening of the Achilles tendon or calf muscles might have a detrimental effect on the loading and energy storage capacity, influencing the propulsion ability and, therefore, influencing the ability to run [25,26]. Conversely, reducing the spasticity of the rectus femoris might be beneficial, as it was identified as a negative predictor for the ability to run [14]. However, due to the unequal group sizes and small case numbers, these hypotheses cannot be reliably analyzed within the current study.

A decreasing strength–weight ratio is one reason why ambulatory patients with CP lose the ability to walk [27]. Therefore, a high BMI might be unfavorable for the ability to run, which is supported by previous results [14]. However, no significant group differences were found for post-surgical BMI deviations, indicating that BMI changes may not be a major factor for the gain or loss of the ability to run after SEMLS.

Despite the fact that surgical interventions aim to improve function [6], it should be mentioned that not every surgery aims to improve the ability to run and that, in some cases, maintaining walking function is a more appropriate goal considering the overall disease severity and functional status of the individual patient.

## 5. Limitations

The unequal group and small sample sizes limited the statistical power of the current study and a more detailed analysis of single surgical procedures or the inclusion of more predictor variables in the logistic regression model was statistically not feasible. The control group was naturally younger and less affected than the surgically treated patients. This difference was statistically addressed by using a matching algorithm and weights in the group comparisons. Despite the remaining unequal preconditions, namely age and GPS, which arguably influence the ability to run, all age groups up to the age of 17 were represented in the control group and 55% were within one SD of the GPS of the surgically treated patients. In addition, the fact that only 1 of the 71 control patients lost the ability to run is a strong indicator that running ability is rarely spontaneously or naturally lost within young conservatively treated people with CP.

Furthermore, besides the restriction of the follow-up measurement time point to a minimum of 12 months, follow-up time points were uncontrolled. Therefore, patients had different time spans to recover from the surgery, which may influence the presence or absence of the ability to run.

The measurements were bound to the gait laboratory with a 13 m runway and running ability was defined by successfully achieving a double float phase during at least one step. While accomplishing a double float phase shows a certain level of strength, coordination and balance, considerable differences concerning the movement quality, running pace and endurance may remain within the patients who were able to run.

Despite these limitations, the study offers unique insights into the presence of the ability to run after SEMLS and associated factors.

## 6. Conclusions

The study shows that objectively assessing the pre-surgical function is an important aspect in personalizing the clinical decision making and could help to optimize the outcome of SEMLS. Pre-surgical function is an important factor for the presence of the ability to run after SEMLS and often defines how many surgical corrections are necessary. In addition, the number of surgical procedures is a significant predictor for the ability to run after surgery. Therefore, careful evaluation and consideration is warranted, especially in patients with a low functional level who are able to run pre-surgery and may, therefore, be at risk of losing the ability.

## Figures and Tables

**Table 1:** Patient characteristics.

Precondition	run00	run01	run10	run11	Control
Group size (N)	30	8	17	43	71
Female/male (%)	40/60	37/63	47/53	35/65	53/47
Age pre	12.3 (3.4)	12.4 (4.4)	12.3 (2.4)	11.6 (3.7)	9.9 (2.6)
BMI pre	18.7 (3.5)	17.6 (2.1)	17.9 (2.8)	17.8 (3.2)	17.1 (2.5)
GMFCS I/II (%)	0/100	0/100	18/82	42/58	65/35
Hemi-/bilateral CP (%)	0/100	33/66	18/82	37/63	28/72
Surgery (uni-/bilateral)	30/70	37/63	41/59	70/30	-

Non-runners (run00); ability gained (run01); ability lost (run10); runners (run11). Age/BMI mean (SD).

**Table 2:** Group means, standard deviations (SD), Kruskal–Wallis and post-hoc test results.

Variables	run00 (a)	run01 (b)	run10 (c)	run11 (d)	Control (e)	Kruskal–Wallis	Post-Hoc Test
Precondition	Mean (SD)	Mean (SD)	Mean (SD)	Mean (SD)	Adj. Mean (SD)	*p*-value	
Age pre-surgery (years)	12.3 (3.4)	12.4 (4.4)	12.3 (2.4)	11.6 (3.7)	11.7 (3.0)	0.810	-
Walking GPS pre (°)	10.58 (2.85)	9.59 (2.68)	10.90 (4.06)	9.03 (2.16)	9.43 (2.04)	0.113	-
Walking velocity (pre) (non-dimensional)	**0.33 (0.06)**	0.37 (0.05)	**0.37 (0.05)**	**0.43 (0.06)**	**0.39 (0.06)**	**<0.001**	**ad, ae, cd**
Difference pre–post							
Walking GPS diff. (°)	−0.69 (2.50)	−1.28 (2.95)	−2.74 (3.27)	−1.41 (2.18)	−0.56 (2.04)	0.187	-
Walking velocity diff. (non-dimensional)	**−0.05 (0.08)**	**0.02 (0.06)**	**−0.08 (0.11)**	**−0.03 (0.06)**	**0.001 (0.05)**	**0.007**	**ab, ae, bc, bd, ce, de**
BMI diff. (% from norm)	0.39 (9.20)	−3.08 (7.03)	1.78 (9.33)	−1.68 (6.75)	−0.29 (5.01)	0.432	-
Post-surgery							
No. surgical procedures	**6.3** **(2.5)**	**3.5** **(0.9)**	**5.9** **(1.7)**	**3.4** **(1.3)**	−	**<0.001**	**ab, ad, bc, cd**
Time to post (month)	25.4 (15.7)	20.5 (7.4)	20.8 (6.8)	21.8 (7.4)	20.8 (9.3)	0.770	-

Bold = significant difference (*p* < 0.05); post-hoc test results: the letter indicates to which group(s) sig. differences exist. Groups: a = Non-runners (run00); b = ability gained (run01); c = ability lost (run10); d = runners (run11); e = control. Adj. mean (adjusted mean based on propensity score weighting).

## Data Availability

The data are not publicly available due to data protection.
